# Effect of a supervised intermittent exercise program on insomnia in breast cancer patients undergoing chemotherapy

**DOI:** 10.1007/s10549-026-07923-7

**Published:** 2026-02-26

**Authors:** Chloé Drozd, Elsa Curtit, Quentin Jacquinot, Pauline Roux, Sophie Paget-Bailly, Valérie Gillet, Nathalie Meneveau, Fabienne Mougin

**Affiliations:** 1 Université Marie et Louis Pasteur, SINERGIES (UR 4662), F-25000 Besançon, France; 2 Université Marie et Louis Pasteur, UFR STAPS, F-25000 Besançon, France; 3 Université Marie et Louis Pasteur, INSERM U1098 RIGHT, F-25000 Besançon, France; 4 Institut Régional Fédératif du Cancer de Franche-Comté, F-25000 Besançon, France; 5 Service d’Oncologie Médicale, CHU Jean Minjoz, F-25000 Besançon, France; 6 Service de Physiologie – Explorations fonctionnelles, CHU Jean Minjoz, F-25000 Besançon, France; 7 Plateforme Nationale Qualité de Vie et Cancer, F-21000 Dijon, France; 8 Unité de méthodologie et de qualité de vie en cancérologie, CHU Jean Minjoz, F-25000 Besançon, France; 9 Centre Médical Santé Sommeil - Ellipse, Association le Don Du Souffle, F-25000 Besançon, France

**Keywords:** Breast cancer, Insomnia, Sleep disorders, Physical training

## Abstract

**Background:**

Patients with localized breast cancer receiving adjuvant chemotherapy often experience sleep disturbances, especially insomnia, which significantly impacts quality of life. This study primarily aimed to evaluate the effects of a 12-week exercise program on insomnia, with secondary outcomes on sleep quality, anxiety/depression, fatigue, pain, and exercise adaptation.

**Methods:**

In this randomized controlled multicenter trial, 20 women with non-metastatic breast cancer and clinically diagnosed insomnia were assigned to a control or training group. The training group underwent a 12-week supervised intermittent aerobic exercise program during chemotherapy. The primary outcome was objective total sleep time; secondary outcomes included insomnia severity, sleep architecture, sleep quality, anxiety/depression, fatigue, pain, and cardiorespiratory capacity. Assessments were performed before chemotherapy (T-1), at baseline (T0), and post-intervention (T3) using polysomnography, actigraphy, validated questionnaires, and a maximal graded exercise test.

**Results:**

The prevalence of clinical insomnia increased from 47% before diagnosis to 71% at T-1, reaching 100% at T0. Total sleep time did not increase after training (p = 0.97), although sleep fragmentation decreased. Clinical improvement was observed in physical and activity-related fatigue. Finally, both submaximal exercise adaptation parameters (power and VO_2_/HR) and maximal parameters (power, VO_2_ peak, VO_2_/HR) significantly improved.

**Conclusions:**

The training did not increase total sleep time, likely due to insomnia’s multifactorial origin. However, training yielded beneficial effects on objective sleep quality and exercise-induced adaptation. Future research is needed to investigate the various etiologies of insomnia to develop tailored and personalized management approaches.

**Clinical Trials Number:** NCT04867096

**Supplementary Information:**

The online version contains supplementary material available at 10.1007/s10549-026-07923-7.

## Introduction

Insomnia is highly prevalent in breast cancer patients, affecting 30–50%, about twice the rate in the general population [1]. It results from tumor biology, treatment side effects, and stress related to diagnosis, therapy, and fear of recurrence [2]. Symptoms include difficulty initiating or maintaining sleep, frequent awakenings, and reduced total sleep time [3–5]. Many patients with pre-existing insomnia report worsening after cancer diagnosis [6]. During chemotherapy, up to 43% meet criteria for insomnia syndrome [7]. Pain, anxiety, fatigue, and depression frequently co-occur, amplifying quality of life decline and risk of chronic insomnia [1, 6, 8, 9]. Consequently, cancer patients face an increased risk of developing chronic insomnia [10, 11]. Pharmacological treatment with hypnotics remains common but is limited by side effects (drowsiness, cognitive impairment, dependence, tolerance) and poor long-term efficacy [12, 13]. Cognitive Behavioral Therapy for Insomnia (CBT-I) is recommended as first-line non-pharmacological management [14, 15]. More recently, exercise has also emerged as an effective supportive strategy, improving physical fitness and psychosocial well-being with minimal risks [16–20]. The 2023 European guideline for the diagnosis and treatment of insomnia specifically suggests that exercise can serve as an adjuvant therapy [21]. Nevertheless, limited research has examined the efficacy of an intermittent aerobic exercise program for cancer-related insomnia during breast cancer treatments. Therefore, the primary aim of this study was to ascertain whether an individualized, 12-week intermittent aerobic exercise training regimen (45 min, three times a week) could alleviate insomnia in non-metastatic breast cancer patients. Secondary objectives were to evaluate, after 12 weeks of treatment, the subjective perception of insomnia, objective sleep architecture and composition, daytime sleepiness, the symptom cluster (fatigue, pain, anxiety/depression), and cardiorespiratory responses to physical exercise.

## Materials and methods

### Study participants and research design

This study employed a randomized, controlled and multicenter trial design, recruiting women with non-metastatic breast cancer from six medical oncology centers in the Franche-Comté region of Eastern France (Clinical Trials: NCT04867096). Comprehensive details of the study design have previously been published elsewhere [22]. Women with insomnia and stage I–III breast cancer scheduled to receive (neo)adjuvant chemotherapy were randomly assigned in a 1:1 ratio to either receive standard oncological care only (Control Group: CG) or standard care supplemented with an exercise program (Training Group: TG). The exercise was initiated between the first and second sequence of their chemotherapy protocol. Inclusion and exclusion criteria are detailed in supplementary materials (Table S1).

### Supervised exercise program

The TG participated in a 12-week supervised intermittent aerobic program (three 45-min sessions/week), starting between the first and second sequence of chemotherapy [22]. Exercise intensity was individually adjusted to maintain the target heart rate, and all sessions were supervised by adapted physical activity specialists at one of four nearby rehabilitation centers.

### Study outcomes

A retrospective assessment was performed prior to cancer diagnosis to document pre-existing insomnia. This time point was not, however, included in the study’s protocol.

Other assessments were conducted at three time points:

- T-1: prior to initiating chemotherapy and the exercise program.
- T0: between the 1 st and 2nd sequences of chemotherapy and before randomization.
- T3: post-exercise program, 3 months after T0.

The primary outcome was total sleep time (TST). The details of all measured variables have previously been described [22].

#### Prior to initiating chemotherapy

**Fig. 1:**
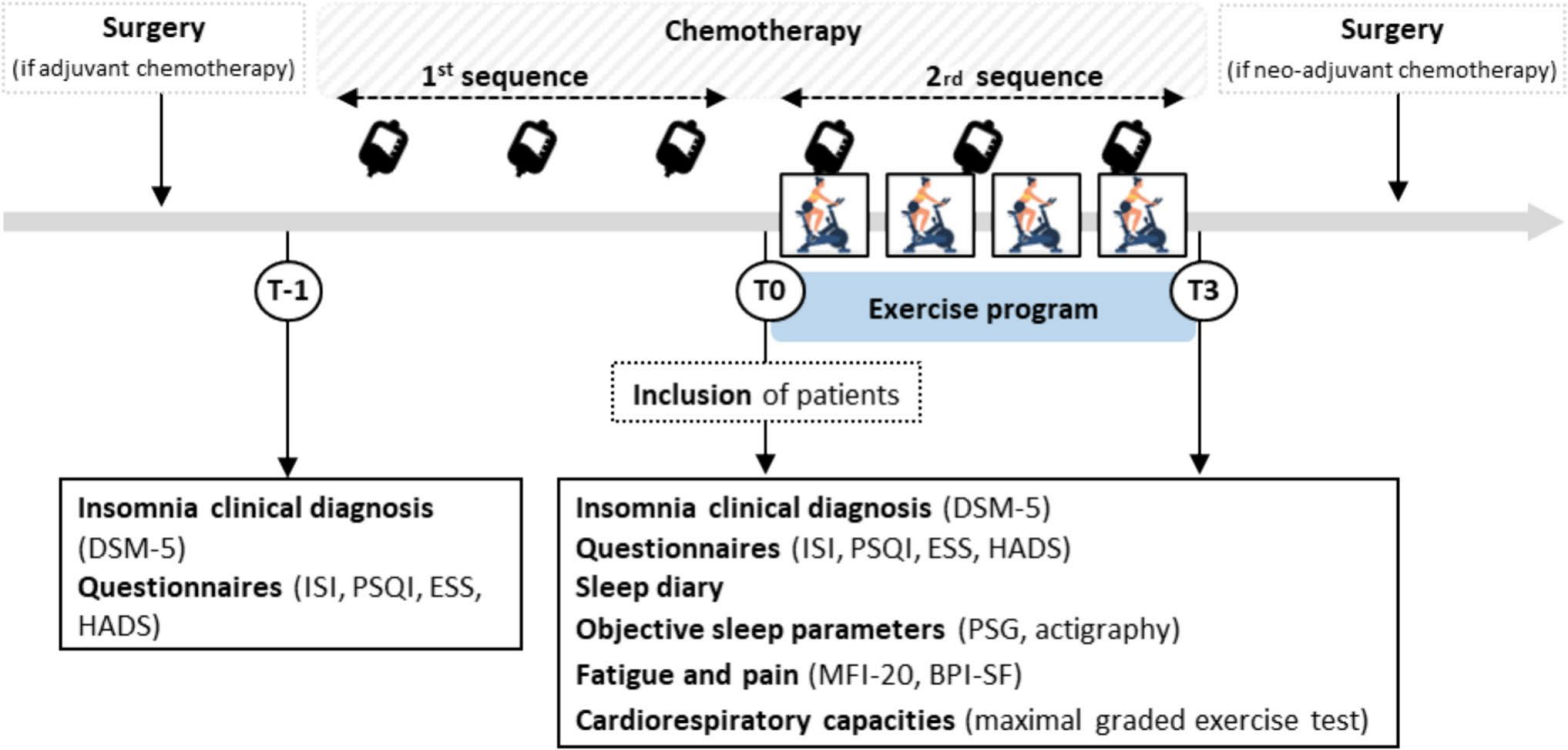
Time and studied variables

Assessment time points are detailed in Fig. 1. All patients underwent a clinical interview based on the DSM-5 to diagnose insomnia and also completed standardized questionnaire: Insomnia Severity Index (ISI), Pittsburgh Sleep Quality Index (PSQI), Epworth Sleepiness Scale (ESS), Hospital Anxiety Depression Scale (HADS). Details of the questionnaires are provided in study protocol [22].

#### During and post-chemotherapy assessments

The aforementioned questionnaires and scales were re-administrated to patients between the first and second sequence of chemotherapy (Fig. 1). In addition, objective sleep was recorded using ambulatory polysomnography (PSG) and analysed according to the American Academy of Sleep Medicine guidelines (Berry et al., 2017). Specific detailed procedures are provided in Drozd, Curtit [22]. The following PSG parameters were assessed: sleep onset latency (SOL), total sleep time (TST), time in bed (TIB), sleep efficiency (TST/TIB × 100), wake after sleep onset (WASO), number and duration of awakenings, sleep stage distribution (N1, N2, N3, REM), sleep quality (Slow Wave Sleep + REM/TST), micro-arousals, periodic limb movements index (PLMI), PLMI with micro-arousals, and apnea–hypopnea index (AHI). Patients also wore an actimeter recorder (ActiGraph wGT3X-BT®) on their non dominant wrist for seven consecutive nights. Only total sleep time (TST) was retained for the present analysis as the main actigraphic outcome. A sleep diary was additionally completed for 2 weeks, in conjunction with actigraphy, to differentiate between time spent awake and time spent attempting to sleep. Fatigue and pain were evaluated by self-reported questionnaire, using MFI-20 and BPI-SF. Cardiorespiratory fitness was assessed via a maximal graded exercise test performed on a cycle ergometer. The detailed procedure has been previously described [22]. The following parameters were continuously recorded and evaluated: power output in watts), oxygen uptake (VO_2_, mL·kg⁻^1^·min⁻^1^), oxygen pulse (VO_2_/HR, mL per beat), and heart rate (HR, beats per minute).

## Statistical analysis

### Sample size

TST was selected as a quantitative indicator of insomnia, objectively measured using PSG.

A one-sided Z-test estimated that 57 patients would be needed to detect a 40-min difference in TST (TG 460 vs CG 420 min) with 80% power at 5% significance (*p* < 0.05). Three interim analyses were planned at 33% and 66% of the total information (with at least 19 and 38 randomized patients, respectively). The present study reports results from the first pre-planned interim analysis.

### Data analysis

Statistical analyses were performed with R (version 4.0.2) and JASP (version 0.19) software.

Descriptive statistics were applied to population, disease and treatment characteristics; sleep data from PSG and actigraphy; ESS and HADS scores; MFI-20 and BPI-SF scores. Qualitative variables are presented as number and percentage, while quantitative variables are described as mean ± standard deviation (SD). For the ISI, PSQI, and MFI-20 questionnaires, a Minimally Clinically Important Difference (MCID) was defined respectively as ≥ 6 points [23], ≥ 3 points [24] and ≥ 2 points.

The comparison of the average TST for the primary endpoint was performed using a one-sided Student t-test. The normality of the continuous variable distributions (including ISI, PSQI scores, cardiorespiratory parameters) was checked using the Shapiro–Wilk test. Student's t-test or Wilcoxon signed-rank test were carried out for paired comparisons. The significance threshold was set at 0.05 for all comparisons.

## Results

### Participants

Between December 2020 and October 2023, among 214 patients screened, 24 were randomized, 12 patients in each group. Two participants in each group discontinued the study and withdrew their consent. Thus, 20 patients completed the study protocol (supplementary materials, Figure S1).

Baseline differences emerged between groups, with women in the training group being younger and more frequently premenopausal (Table 1).

**Table 1:** Baseline characteristics of patients

	Control group	Training group
Patient characteristics		
Age, years	49.4 ± 9.3	44.1 ± 6.1
Menopausal women		
Yes	3 (30)	0 (0)
No	7 (70)	9 (100)
Missing data	0	1
BMI, kg/m^2^, n (%)		
Underweight (< 18.5)	1 (10)	1 (10)
Normal weight (18.5–25)	6 (60)	4 (40)
Overweight (25–30)	3 (30)	5 (50)
Disease characteristics		
SBR grade		
1	0 (0)	1 (11.1)
2	5 (50)	5 (55.6)
3	5 (50)	3 (33.3)
Missing data	0	1
Histological type		
Invasive ductal carcinoma	10 (100)	10 (100)
Cancer type		
HER2 + HR +/HR-	5 (50)	2 (20)
HER2- HR +	2 (20)	7 (70)
Triple-negative	3 (30)	1 (10)
Treatments		
Breast surgery		
Mastectomy	2 (20)	4 (40)
Conservative surgery	8 (80)	6 (60)
Axillary surgery		
Sentinel node	8 (80)	10 (100)
Axillary dissection	2 (20)	1 (10)
Chemotherapy		
Adjuvant	5 (50)	8 (80)
Neoadjuvant	5 (50)	2 (20)
Chemotherapy protocol		
EC + Docetaxel	5 (50)	5 (50)
EC + Paclitaxel	1 (10)	5 (50)
Paclitaxel-Carboplatin + EC	4 (40)	0 (0)
EC frequency		
Every 2 weeks	0 (0)	4 (40)
Every 3 weeks	10 (100)	6 (60)

*BMI* body mass index, *EC* epirubicin cyclophosphamide

### Insomnia and co-occurring symptoms from diagnosis to chemotherapy initiation

Among the 20 patients included, 19 completed the full DSM-5 clinical interview prior to diagnosis at T-1 and T0. In contrast, only 14 patients completed the entire set of self-reported questionnaires (ISI, PSQI, ESS, and HADS) at T-1 and T0.

#### DSM-5-based clinical assessment of insomnia

**Fig. 2:**
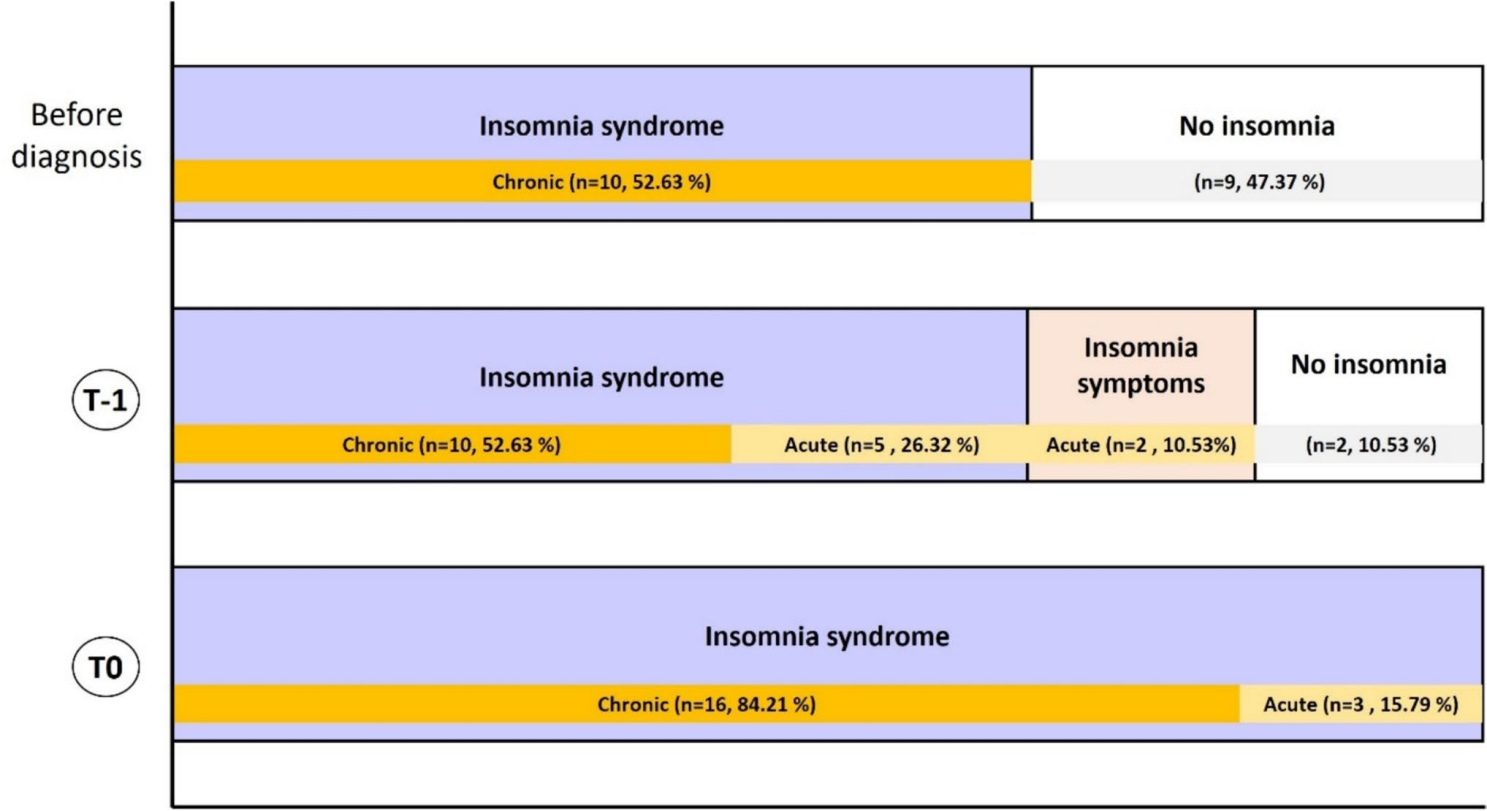
Clinical insomnia with DSM-5 before diagnosis, at T-1 and T0

Prior to the cancer diagnosis, 52% of patients already exhibited chronic insomnia syndrome. At T-1, this proportion rose to 78% (52% with chronic insomnia syndrome and 26% with acute insomnia syndrome), with an additional 10% reporting occasional symptoms. At T0, 84% of patients had developed chronic insomnia, and 15% represented new cases of acute insomnia syndrome appeared (Fig. 2).

#### Insomnia severity

At T-1, 30% of patients had no insomnia, whereas at T0 all scored ISI ≥ 8, with “mild insomnia” rising from 30 to 35%, moderate cases nearly doubled (30% to 57%), and “severe insomnia” remained stable, with one patient at T-1 and T0 (Table 2).

**Table 2:** Course of insomnia, sleep quality, daytime sleepiness, anxiety and depression scores before and during chemotherapy (T-1 *vs* T0)

	Patients (n = 14)	
	T-1	T0
ISI		
Absence of insomnia (≤ 7)	4 (30.77)	0 (0)
Mild subclinical insomnia (8–14)	4 (30.77)	5 (35.71)
Moderate subclinical insomnia (15–21)	4 (30.77)	8 (57.14)
Severe subclinical insomnia (≥ 22)	1 (7.69)	1 (7.14)
Missing data	1	0
PSQI		
“Good sleeper” (≤ 5)	1 (10)	2 (14.29)
“Poor sleeper” (> 5)	13 (90)	12 (85.71)
Missing data	0	0
ESS		
Absence of sleepiness (≤ 8)	11 (78.57)	9 (64.29)
Mild daytime sleepiness (9–14)	3 (21.43)	4 (28.57)
Severe daytime sleepiness (≥ 15)	0 (0)	1 (7.14)
Missing data	0	0
HADS anxiety		
Absence of symptoms (≤ 7)	9 (64.29)	8 (57.14)
Mild symptoms (8–10)	1 (7.14)	3 (21.43)
Moderate symptoms (≥ 11)	4 (28.57)	3 (21.43)
Missing data	0	0
HADS depression		
Absence of symptoms (≤ 7)	4 (28.57)	12 (85.71)
Mild symptoms (8–10)	1 (7.14)	1 (7.14)
Moderate symptoms (≥ 11)	9 (64.29)	1 (7.14)
Missing data	0	0

*ESS* epworth sleepiness scale, *HADS* hospital anxiety and depression scale, *ISI* insomnia severity index, *PSQI* pittsburgh sleep quality index

#### Sleep disorders and sleep quality

Most patients were classified as “poor sleepers” (PSQI scores > 5) at T-1 (90% of patients) and T0 (85% of patients).

#### Sleepiness

Mild daytime sleepiness affected few patients from T0 (21%) to T3 (28%). No patients experienced severe sleepiness at T-1, but one patient did at T0.

#### Anxiety and depression

Anxiety remained stable, with a majority of patients being symptom-free à T-1 and T0 (64% *vs* 57%), and only slight shifts observed between mild and moderate symptoms. Depression decreased: 64% patients with moderate symptoms at T-1 dropping to only one patient at T0, and most became symptom-free (85%).

### Insomnia, sleep architecture, symptom cluster and cardiorespiratory variables during the exercise intervention period

#### Insomnia based on total sleep time

Due to the low number of patients who underwent PSG at T3 (12 of 20; 8 in the CG and 4 in the TG), the robustness of analyses using this method was limited. Consequently, actigraphy values were used to replace missing PSG data, with a linear regression model confirming a significant correlation between PSG and actigraphy results (*p* = 0.038) to support this decision. In the TG, TST unexpectedly decreased, with a mean difference of − 63.38 min (95% CI − 124.5; − 2.26), from 07:16 ± 00:35 at T0 to 06:13 ± 01:13 at T3; while it remained stable in the CG, with a mean difference of + 12.5 min (95% CI − 26; 44), from 06:59 ± 00:51 min at T0 *vs* 07:08 ± 00:49 at T3. After the exercise program, no between-group difference was observed (*p* = 0.97). Accordingly, the proportion of patients with TST ≤ 7 h rose to 70% in the TG *vs* 60% in the CG (Supplementary Table S2).

#### Composition and sleep architecture

The TG showed a reduction in sleep latency (–7 min) whereas the CG showed an increase (+ 4 min). Changes in sleep efficiency were similar between groups (TG: + 4.9%; CG: + 4.1%). N3 sleep stage increased slightly in both groups (Table 3). The number of micro awakenings decreased more in the TG (–27 *vs* –19). WASO decreased in both groups at T3 (TG: 34 ± 11.4 min *vs* 55.10 ± 26.6 min; CG: 61 ± 2.1 min *vs* 86.7 ± 65.8 min). Sleep apnea disappeared in the TG but persisted among four patients in the CG at T3. Additionally, only one patient in the CG had PLMs at T3.

**Table 3:** Data from PSG at T0 and T3 in control and training groups

	Control group		Training group	
	T0	T3	T0	T3
Sleep variables				
Sleep onset time, h:min	23:29	23:11	23:07	22:48
Wake-up time, h:min	08:02	07:11	07:19	06:16
SOL, min	25.3 ± 15.9	29.5 ± 31.5	24 ± 27.3	17.4 ± 24.2
TST, h:min	06:59 ± 00:51	07:11 ± 00:55	07:16 ± 00:35	06:51 ± 00:39
TIB, h:min	09:13 ± 40	08:49 ± 109	08:43 ± 22	07:49 ± 58
SE, %	76 ± 9.6	80.1 ± 10.2	83.4 ± 6.5	88.3 ± 4.5
Sleep quality, %	49.4 ± 11.1	50.5 ± 13.5	45.5 ± 8	45.7 ± 6.6
Number of awakenings	36. ± 14.7	32.9 ± 5.6	26.8 ± 10.2	23.3 ± 12.1
Number of micro awakenings	124.7 ± 33.2	105.9 ± 38.7	140.9 ± 71.9	113.8 ± 48.5
WASO, min	86.7 ± 65.8	61.2 ± 35.1	55.1 ± 26.6	34 ± 11.4
Sleep architecture, %				
Stage N1	11.9 ± 5.02	8.6 ± 2.3	10.8 ± 3.9	8.6 ± 3.9
Stage N2	38.7 ± 10.3	40.9 ± 12.6	43.8 ± 8.2	45.7 ± 8.8
Stage N3	26.5 ± 9.8	28.6 ± 10.3	21 ± 7.1	23.5 ± 6.5
Stage REM	22.8 ± 7.5	22 ± 6.4	24.4 ± 3.4	22.3 ± 3.5
Periodic limb movements, n (%)				
Presence of PLM ≥ 15/h	1 (10)	1 (12.5)	1 (10)	0 (0)
PLM with micro awakenings > 5/h				
Respiration, n (%)				
Absence of SAS, IAH < 5/h	5 (50)	4 (50)	8 (80)	4 (100)
Mild SAS, 5/h ≤ IAH < 15/h	3 (30)	4 (50)	1 (10)	0 (0)
Moderate SAS, 15/h ≤ IAH < 30/h	1 (10)	0 (0)	1 (10)	0 (0)
Severe SAS, IAH ≥ 30	1 (10)	0 (0)	0 (0)	0 (0)
Missing data	0	2	0	6

Values are presented as mean ± standard deviation for continuous data and as number (%) for categorical data.

*PLMs* Periodic Limb Movements, *SAS* Sleep Apnea Syndrome, *SE* Sleep Efficiency, *SOL* Sleep Onset Latency, *TIB* Time In Bed, *TST* Total Sleep Time, *WASO* Wakefulness After Sleep Onset

#### Subjective insomnia

For the ISI score, no significant improvement was observed in the TG and mild insomnia persisted (14.9 ± 5.1 *vs* 12.8 ± 8.1) (mean difference: 2.7; 95% CI − 4.5; 8.5). In the CG, the score decreased from moderate (15.3 ± 3.3) to mild insomnia (12.5 ± 5.5) (mean difference: 2.5; 95% CI 2.5; − 2; 9) (Supplementary Table S3). Only two patients in each group achieved the MCID (≥ 6 points).

#### Subjective quality and sleep disorders

PSQI score did not significantly change in the training (9.8 ± 4 at T3 *vs* 10.5 ± 4.8 at T0; mean difference: −1; 95% CI − 7.5 to 5) and control groups (9.6 ± 2.8 at T3 *vs* 6.9 ± 2.6 at T0; mean difference: − 3; 95% CI − 5.5 to 0) (Supplementary Table S3). The MCID reduction (≥ 3) was reached by 3 patients in the TG and 5 in the CG.

#### Symptom cluster of anxiety/depression, daytime sleepiness, and fatigue

The analysis of symptom burden highlighted different trajectories between groups.

In the CG, anxiety stayed mild (7.9 ± 4.1 to 8.7 ± 3.8), whereas in the TG, it rose from absence of symptoms (6.9 ± 3.7) to mild anxiety in post-exercise (9.8 ± 4.5). Depressive symptoms stayed below 5 in both groups. Daytime sleepiness score showed no significant change.

The pain severity score decreased in the CG (4.9 ± 3.7 to 2.2 ± 3.3) but remained unchanged in the TG (2.3 ± 2.4 to 2.4 ± 2.7). Pain interference was unchanged in the CG (1.6 ± 2.2 to 1.5 ± 2.0) whereas in the TG, it slightly increased (0.9 ± 2.2 to 1.5 ± 2.7) (Table 4).

**Table 4:** Scores of anxiety/depression, sleepiness and pain from T0 to T3 in control and training groups

	Control group		Training group	
	T0	T3	T0	T3
HADS				
Anxiety	7.9 ± 4.1	8.7 ± 3.8	6.9 ± 3.7	9.8 ± 4.5
Depression	4.6 ± 2.6	4.1 ± 4.6	4.7 ± 2.3	4.3 ± 3.6
ESS	7.2 ± 4.4	6.6 ± 3.9	6.5 ± 3.4	5.8 ± 3.8
BPI-SF				
Pain severity	4.9 ± 3.7	2.2 ± 3.3	2.3 ± 2.4	2.4 ± 2.7
Pain interference	1.6 ± 2.2	1.5 ± 2.0	0.9 ± 2.2	1.5 ± 2.7

Values are presented as mean ± standard deviation.

*HADS* hospital anxiety and depression scale, *ESS* epworth sleepiness scale, *BPI-SF* brief pain inventory—short form

Regarding fatigue assessed with the MFI-20 and completed by 10 patients from the CG and 8 patients from the TG, the TG showed clinical improvements in “Physical Fatigue” and “Reduced Activity”, with 3 out of 8 patients improving and 3 remaining stable in each. In contrast, the CG improved mainly in “Reduced Motivation” (4 patients improved, 6 stable) but also recorded more frequent deterioration in “Physical Fatigue” (3 out of 10 patients). Overall, the CG exhibited a greater number of patients who experienced deterioration across various MFI-20 sub-dimensions. Detailed individual variations in MFI-20 subscales are provided in the supplementary materials (Figure S2).

#### Cardiorespiratory variables

At T3, the TG demonstrated a significant increase in workload at both the first (VT1) (mean difference: 17.1 W; 95% CI 4.7; 29.49) and second ventilatory threshold (VT2) (mean difference: 20.5 W; 95% CI 5.84; 35.16), as well as at maximal power output (mean difference: 20.4 W; 95% CI 4.81; 35.99) (Table 5).

**Table 5:** Changes in physiological parameters at VT_1_, VT_2_, and peak exercise in control and training groups

		Control group			Training group		
		T0	T3	T3–T0 (95% CI)	T0	T3	T3–T0 (95% CI)
VT_1_	Power, watts	58.5 ± 11.6	55 ± 13.2	−3.5 (−12.93; 5.93)	76 ± 19.5	93.1 ± 19.2	17.1 (4.7; 29.49)
	VO_2_, mL.min^−1^.kg^−1^	12.7 ± 2.9	11.7 ± 2.4	−0.97 (−2.89; 0.95)	15.1 ± 2.6	17.9 ± 3.6	2.77 (0.05; 5.49)
	VO_2_/HR, mL.bpm^−1^	6.4 ± 1.5	6.2 ± 1.4	−0.25 (−1.07; 0.57)	7.7 ± 1.3	9.2 ± 2.1	1.54 (0.6; 2.47)
	HR, beat.min^−1^	126.5 ± 17.8	123.3 ± 16.9	−3.2 (−13.70; 7.3)	129.4 ± 10.8	132.2 ± 13.8	2.8 (−7.42; 13.02)
VT_2_	Power, watts	82.8 ± 13.4	84 ± 13.7	1.2 (−9.64; 12.04)	104.9 ± 24.7	125.4 ± 17.1	20.5 (5.84; 35.16)
	VO_2_, mL.min^−1^.kg^−1^	16.5 ± 3.6	16.5 ± 2.7	−0.02 (−1.82; 1.78)	20.3 ± 4	23.2 ± 3.5	2.23 (−0.42; 4.88)
	VO_2_/HR, mL.bpm^−1^	7.3 ± 1.3	7.2 ± 1.5	−0.17 (−1.05; 0.71)	8.8 ± 1.6	10 ± 1.4	1.15 (−0.29; 2.01)
	HR, beat.min^−1^	144.5 ± 21.5	149.4 ± 18.7	4.9 (−5.54; 15.34)	155.4 ± 11.7	156.7 ± 12.7	1.3 (−6.06; 8.66)
Peak	Power, watts	96.5 ± 17.3	99 ± 15.1	2.5 (−4.08; 9.08)	126.9 ± 31.2	147.3 ± 21.8	20.4 (4.81; 35.99)
	VO_2_, mL.min^−1^.kg^−1^	21.2 ± 4.2	21.2 ± 4.3	0.06 (−1.82; 1.94)	25.8 ± 4.5	28.5 ± 3.5	2.66 (−0.02; 5.35)
	VO_2_/HR, mL.bpm^−1^	8.7 ± 1.8	8.4 ± 1.4	−0.31 (−0.97; 0.35)	9.8 ± 1.4	12 ± 3.4	2.16 (−0.26; 4.06)
	HR, beat.min^−1^	156.8 ± 25.2	161.4 ± 19	−4.6 (−2.66; 11.86)	172.4 ± 12.4	173.6 ± 13.7	1.2 (−7.03; 9.43)

Values are expressed as mean ± standard deviation. 95% confidence interval (CI) of the difference between pre- and post-intervention values.* HR* heart rate, *VT*_*1*_ first ventilatory threshold, *VT*_*2*_ second ventilatory threshold.

VO_2_ also significantly improved at VT_1_ (mean difference: 2.77 mL·min⁻^1^·kg⁻^1^; 95% CI 0.05; 5.49). Furthermore, oxygen pulse (VO_2_/HR) was higher at VT_1_, VT_2_, and maximal exercise compared to baseline (mean differences: 1.54 mL·min⁻^1^·kg⁻^1^,95% CI 0.6; 2.47; 1.15 mL·min⁻^1^·kg⁻^1^, 95% CI − 0.29; 2.01; 2.16 mL·min⁻^1^·kg⁻^1^, 95% CI, − 0.26; 4.06, respectively), indicating enhanced oxygen utilization per heartbeat. In contrast, CG showed no significant changes in workload, VO₂ or VO2/HR after three months.

#### Adherence and progression in the exercise program

Patients completed an average of 31 ± 3 out of 36 prescribed exercise sessions, resulting in an adherence rate of 86.1%.

## Discussion

In this randomized controlled trial, the supervised intermittent exercise program did not increase total sleep time. However, the intervention reduced sleep fragmentation, improved specific dimensions of fatigue, and enhanced exercise capacity.

### Diagnosis and treatment effects on insomnia and associated sleep disorders

While the relationship between sleep characteristics and survival in breast cancer remains uncertain and complex [25], understanding the etiologies of sleep disturbances in cancer patients is crucial for optimizing their management. Unlike most studies conducted after treatment, our results showed that nearly 60% of patients already had chronic insomnia before diagnosis, a much higher rate than that reported by Fleming, Randell [26], who observed considerably lower pre-diagnosis insomnia rates (8.1% for insomnia syndrome and 16.8% for symptoms). These findings suggest that future studies should investigate whether initiating insomnia management earlier, ideally before chemotherapy, could yield greater effects.

The anxiety associated with a breast cancer diagnosis, compounded by uncertainty regarding treatment and anticipated body changes, likely contributes significantly to sleep disruption. In our study, 79% of patients exhibited insomnia syndrome and 10.5% reported insomnia symptoms post-diagnosis, rates higher than those reported by Fleming, Randell [26]. This acute increase aligns with prior research indicating that cancer-related hypervigilance and maladaptive sleep beliefs contribute to persistent sleep difficulties. Stress-related hyperactivation of the hypothalamic–pituitary–adrenal axis likely promotes nocturnal awakenings and delayed sleep onset, reinforcing a vicious cycle of poor sleep-anxiety.

Similarly, 84% of our patients experienced insomnia during chemotherapy, compared to only 24% of patients reported by Fleming, Randell [26]. Savard, Villa [27] observed lower rates in other cancers, with insomnia declining two months post-diagnosis. Factors such as female sex, reactive personality traits, anxiety, or surgery may explain higher rates in our sample. Chemotherapy and corticosteroids may further impair sleep via neuroinflammation, melatonin disruption, or hyperarousal [28, 29].

In our study, ISI scores indicated that 30.8% of patients had subclinical insomnia before diagnosis, increasing to 100% by mid-chemotherapy, with a two-fold rise in moderate cases. Before treatment, 90% were already classified as “poor sleepers,” according to PSQI, a status unchanged throughout therapy. Gonzalez, Eisel [30] and Sanford, Wagner [31] reported lower rates (57.5% and 65.8% respectively) before treatment. Sleep disturbances during chemotherapy likely result from multiple interacting factors, including age, menopausal status, and other clinical variables [32], making it challenging to isolate chemotherapy’s specific impact on insomnia.

### Effect of exercise program on insomnia and related disorders

Our results showed no increase in TST after three months of training, whether measured by PSG or actigraphy. This may reflect a ceiling effect, since baseline TST was already high: only 20% of patients had < 7 h at T0. Thus, most of participants were already within the 7–9 h range recommended by the National Sleep Foundation, leaving limited room for further improvement. This result must be interpreted cautiously due to the limited number of PSG recordings available at T3.

Despite unchanged TST, sleep efficiency improved, nocturnal awakenings decreased, and WASO was reduced, indicating less fragmented sleep. Self-perceived sleep disturbances (*e.g.*, PSQI, ISI) did not improve significantly, and complaints were not accompanied by excessive daytime sleepiness, suggesting patients may tolerate or underreport the daytime consequences of poor sleep. Previous studies reported mixed finding: Wang, Boehmke [33] observed reduced PSQI scores after a six-week walking program, though insufficient to resolve sleep disturbances; while Rubio-Arias, Marín-Cascales [34] found that regular aerobic exercise for 12–16 weeks reduced PSQI scores in healthy women but not insomnia severity. In cancer patients, variations in training duration (4 to 24 weeks) could explain inconsistent results. Longer or earlier interventions (*e.g.*, at chemotherapy onset) might yield stronger effects.

Insomnia pathophysiology involves hypervigilance and neurobiological dysregulation [35]. This model links psychological aspects of insomnia (*e.g.,* hypervigilance) with neurobiological mechanisms involving the prefrontal cortex and limbic system. A key feature is somatic hyperarousal, with persistent activation of somatic, cognitive, and cortical systems at night, impairing both sleep onset and quality.

Therefore, the exercise program alone may have had limited efficacy in downregulating the heightened arousal mechanisms underlying sleep initiation and maintenance difficulties in a subset of this population.

Regarding the symptom cluster, anxiety slightly increased in the TG, possibly due to sympathetic activation. While physical fatigue and reduced activity dimensions improved clinically. Future interventions could consider incorporating relaxation techniques, such as mindfulness or breathing exercises, or integrating Cognitive Behavioral Therapy for Insomnia (CBT-I) alongside exercise, though challenging, could potentially reduce anxiety and further improve sleep. Although exercise is widely recognized as an effective treatment for cancer-related fatigue, our study did not demonstrate a decrease in overall fatigue compared to pre-training levels. This might be explained by the overlap between exercise-induced fatigue and cancer-related fatigue, making result interpretation more complex, especially as patients may conflate these two forms of fatigue. Further research is warranted to elucidate this distinction.

### Effect of exercise program on physiological responses to exercise

Our study demonstrates that the training program significantly improved both submaximal and maximal exercise capacity. It also exerted a positive effect on cardiac response, as submaximal HR remained unchanged despite increased workload. Maximal HR approached to theoretical maximum, confirming effort to exhaustion. Aerobic capacity improved, evidenced by a shift in ventilatory thresholds, reflected by an increase in submaximal VO_2_ for higher workloads. This enhanced aerobic capacity and VO_2_ peak contributed to reducing physical deconditioning, a major consequence of cancer-related fatigue. Maintaining or improving cardiorespiratory fitness during treatment is crucial, as it may contribute to better treatment tolerance and post-treatment recovery. Although VO_2_ peak improved after training, reaching values comparable sedentary women of the same age, it remained below the levels observed in healthy, active women aged 50 [≈32.02 mL/kg/min according to Fitzgerald, Tanaka [36]. These findings align with a previous study by our team, which employed a similar training protocol for HER2 + breast cancer patients treated with trastuzumab [18], demonstrating that the program was well-tolerated with high patient adherence. Our results are comparable to those of Isanejad, Nazari [37], who observed a VO_2_ peak increase of + 3.61 mL/kg/min after a similar 12-week intermittent training program. The slightly smaller improvement in our study (+ 2.7 mL/kg/min) may be attributed to our retraining protocol being conducted concurrently with chemotherapy, whereas Isanejad's study was conducted one month after treatment, during hormone therapy. Additionally, our patients exhibited lower pre-training VO_2_ values, potentially reflecting higher baseline deconditioning. Similar findings were reported by Dolan, Campbell [38] in post-adjuvant therapy following high-intensity interval training (50–90% of VO_2_ max) or continuous training (55–70% of VO_2_ max) after 18 sessions over 6 weeks.

### Strengths and limits

Our study offers unique contributions by simultaneously evaluating both objective and subjective sleep disturbances, particularly insomnia during chemotherapy, when patients are most vulnerable. The use of ambulatory polysomnography during active chemotherapy represents a rare and important methodological strength. This rigor is complemented by diligent application of validated clinical tools to assess sleep and insomnia, thus minimizing potential measurement biases. While previous studies have explored the impact of physical activity on various treatment side effects, few have specifically investigated sleep during this period. Furthermore, intermittent training, despite its recommendations for various conditions, has rarely been applied to chemotherapy patients experiencing insomnia. The study also included a detailed assessment of cardiopulmonary adaptations, confirming both the physiological benefits and the overall feasibility and safety of supervised interval training offering critical guidance for future clinical implementation.

The sample was substantially underpowered, making this interim analysis exploratory and limiting the interpretability of the primary outcome. Additionally, the baseline imbalance with women in the training group being younger and more frequently premenopausal, represents a potential confounder that may influenced sleep-related outcomes. While Total Sleep Time (TST) was the designated primary outcome, sleep quality may represent an equally relevant clinical parameter. Future research should, therefore, incorporate both patient-reported and objective measures for a more assessment of exercise effect on sleep.

## Conclusion

Although the supervised exercise program did not significantly increase extend TST in breast cancer patients undergoing chemotherapy, it may have enhanced sleep continuity, reduced certain dimensions of fatigue, and substantially improved physiological fitness. These exploratory findings underscore the need for larger, adequately powered randomized controlled trials and suggest that exercise holds potential as a valuable component within multimodal insomnia management strategies for this population.

## Supplementary Information

Below is the link to the electronic supplementary material.

Supplementary file1 (DOCX 22 KB)

Supplementary file2 (DOCX 122 KB)

## Data Availability

No datasets were generated or analysed during the current study.

## Abbreviations

AHI: Apnea–hypopnea index
BPI-SF: Brief pain inventory short form
CBT-I: Cognitive behavioral therapy for insomnia
CG: Control group
DSM-5: Diagnostic and statistical manual of mental disorders 5th edition
EC: Epirubicin cyclophosphamide
ECG: Electrocardiogram
EEG: Electroencephalogram
ESS: Epworth sleepiness scale
HADS: Hospital anxiety depression scale
HR: Heart rate
ISI: Insomnia severity index
MFI-20: Multidimensional Fatigue Inventory 20
NSF: National sleep foundation
PLMI: Periodic limb movement index
PSG: Polysomnography
PSQI: Pittsburgh sleep quality index
QoL: Quality of life
REM: Rapid eye movement
RPE: Rating of perceived exertion
SD: Standard deviation
SE: Sleep efficiency
SOL: Sleep onset latency
TG: Training group
TIB: Time in bed
TST: Total sleep time
VO2: Oxygen consumption
VO2 Peak: Peak oxygen uptake
VT1 and VT2: Ventilatory thresholds 1 and 2
WASO: Wake after sleep onset
