# Supplementary material for: Effect of a supervised intermittent exercise program on insomnia in breast cancer patients undergoing chemotherapy
Source: Breast Cancer Res Treat. 2026 Feb 26;216(2):20. doi: 10.1007/s10549-026-07923-7 (PMC12945910; doi:10.1007/s10549-026-07923-7)
Supplement: Supplementary file 1 — Supplementary file1 (DOCX 22 KB) [file 10549_2026_7923_MOESM1_ESM.docx]

**Effect of a supervised intermittent exercise program on insomnia in breast cancer patients undergoing chemotherapy**

Chloé Drozd ^ab*^, Elsa Curtit ^cde^, Quentin Jacquinot ^ad^, Pauline Roux^f^, Sophie Paget-Bailly ^cgh^, Valérie Gillet ^i^, Nathalie Meneveau ^de^ and Fabienne Mougin ^ab^

^a^ Université Marie et Louis Pasteur, SINERGIES (UR 4662), F-25000 Besançon, France.

^b^ Université Marie et Louis Pasteur, UFR STAPS, F-25000 Besançon, France.

^c^ Université Marie et Louis Pasteur, INSERM U1098 RIGHT, F-25000 Besançon, France.

^d^ Institut Régional Fédératif du Cancer de Franche-Comté, F-25000 Besançon, France.

^e^ Service d’Oncologie médicale, CHU Jean Minjoz, F-25000 Besançon, France.

^f^ Service de Physiologie – Explorations fonctionnelles, CHU Jean Minjoz, F-25000 Besançon, France.

^g^ Plateforme Nationale Qualité de Vie et Cancer, F-21000 Dijon, France

^h^ Unité de méthodologie et de qualité de vie en cancérologie, CHU Jean Minjoz, F-25000 Besançon, France

^i^ Centre Médical Santé Sommeil - Ellipse, Association le Don Du Souffle, F-25000 Besançon, France.

* Corresponding author: chloe.drozd@hotmail.fr

**Supplementary materials**

**Table S1. Inclusion and exclusion criteria**

| **Inclusion criteria** | **Exclusion criteria** |
| --- | --- |
| - Women aged >18 and ≤65 years - Histologically confirmed early breast cancer - Undergoing first adjuvant or neoadjuvant sequential chemotherapy with anthracyclines and taxanes - Clinically diagnosed insomnia - No contraindication to performing physical activity - Appropriate contraception use or postmenopausal status | - Presence of metastases - Treatment with melatonin or hypnotics Absence of insomnia diagnosis - Documented depression - Resting oxygen saturation (SaO₂) ≤ 92% - Autoimmune disease (systemic lupus erythematosus, rheumatoid arthritis) - Symptomatic osteoarthritis, cardiovascular disease (angina or uncontrolled hypertension), or lung disease (COPD) - Malnutrition (BMI <18 kg/m²) or >10% weight loss within the past 3 months Psychiatric or cognitive disorders - Pregnant or breastfeeding women |

**Table S2. Changes in TST and proportion of patients with TST ≤ 7h from T0 to T3 in control and training groups**

|  | **Control group** | | | | **Training group** | | | |
| --- | --- | --- | --- | --- | --- | --- | --- | --- |
|  | **T0** | **T3** | **95% CI ^b^** | ***p* value^a^** | **T0** | **T3** | **95% CI ^b^** | ***p* value^a^** |
| **TST (h:min) ^§^** | 06:59 ± 00:51 | 07:08 ± 00:49 | 12.5 (-26; 44) | 0.359 | 07:16 ± 00:35 | 06:13 ± 01:13 | -63.38 (-124.5; -2.26) | 0.044 |
| **TST ≤ 7h, n ^§^** | 5 | 6 | – | – | 2 | 7 | – | – |

Values are presented as mean ± standard deviation.

TST: Total Sleep Time.

**^§^** TST derived from combined PSG at T3 (n=12) and actigraphy data (n=8)

^a^ p values were obtained from paired Student's t-test or Wilcoxon signed-rank test to assess within-group changes over time (pre *vs*. post).

^b^ 95% confidence interval (CI) of the difference between pre- and post-intervention values.

Table S3. Changes in ISI and PSQI scores from T0 to T3 in control and training groups

|  | **Control group** | | | | **Training group** | | | |
| --- | --- | --- | --- | --- | --- | --- | --- | --- |
|  | **T0** | **T3** | **95% CI ^b^** | ***p* value^a^** | **T0** | **T3** | **95% CI ^b^** | ***p* value^a^** |
| **ISI** | 15.3 ± 3.3 | 12.5 ± 5.5 | 2.5 (-2; 9) | 0.340 | 14.9 ± 5.1 | 12.8 ± 8.1 | 2.7 (-4.5; 8.5) | 0.695 |
| **PSQI** | 9.6 ± 2.8 | 6.9 ± 2.6 | -3 (-5.5; 0) | 0.051 | 9.8 ± 4 | 10.5 ± 4.8 | -1 (-7.5; 5) | 0.674 |

Values are presented as mean ± standard deviation.

ISI: Insomnia Index Severity, PSQI: Pittsburgh Sleep Quality Index.

^a^ p values were obtained from paired Student's t-test or Wilcoxon signed-rank test to assess within-group changes over time (pre vs. post).

^b^ 95% confidence interval (CI) of the difference between pre- and post-intervention values.
