# Supplementary material for: Effect of a supervised intermittent exercise program on insomnia in breast cancer patients undergoing chemotherapy
Source: Breast Cancer Res Treat. 2026 Feb 26;216(2):20. doi: 10.1007/s10549-026-07923-7 (PMC12945910; doi:10.1007/s10549-026-07923-7)
Supplement: Supplementary file 2 — Supplementary file2 (DOCX 122 KB) [file 10549_2026_7923_MOESM2_ESM.docx]

**Effect of a supervised intermittent exercise program on insomnia in breast cancer patients undergoing chemotherapy**

Chloé Drozd ^ab*^, Elsa Curtit ^cde^, Quentin Jacquinot ^ad^, Pauline Roux^f^, Sophie Paget-Bailly ^cgh^, Valérie Gillet ^i^, Nathalie Meneveau ^de^ and Fabienne Mougin ^ab^

^a^ Université Marie et Louis Pasteur, SINERGIES (UR 4662), F-25000 Besançon, France.

^b^ Université Marie et Louis Pasteur, UFR STAPS, F-25000 Besançon, France.

^c^ Université Marie et Louis Pasteur, INSERM U1098 RIGHT, F-25000 Besançon, France.

^d^ Institut Régional Fédératif du Cancer de Franche-Comté, F-25000 Besançon, France.

^e^ Service d’Oncologie médicale, CHU Jean Minjoz, F-25000 Besançon, France.

^f^ Service de Physiologie – Explorations fonctionnelles, CHU Jean Minjoz, F-25000 Besançon, France.

^g^ Plateforme Nationale Qualité de Vie et Cancer, F-21000 Dijon, France

^h^ Unité de méthodologie et de qualité de vie en cancérologie, CHU Jean Minjoz, F-25000 Besançon, France

^i^ Centre Médical Santé Sommeil - Ellipse, Association le Don Du Souffle, F-25000 Besançon, France.

* Corresponding author: [chloe.drozd@hotmail.fr](mailto:chloe.drozd@hotmail.fr)

**Supplementary materials**


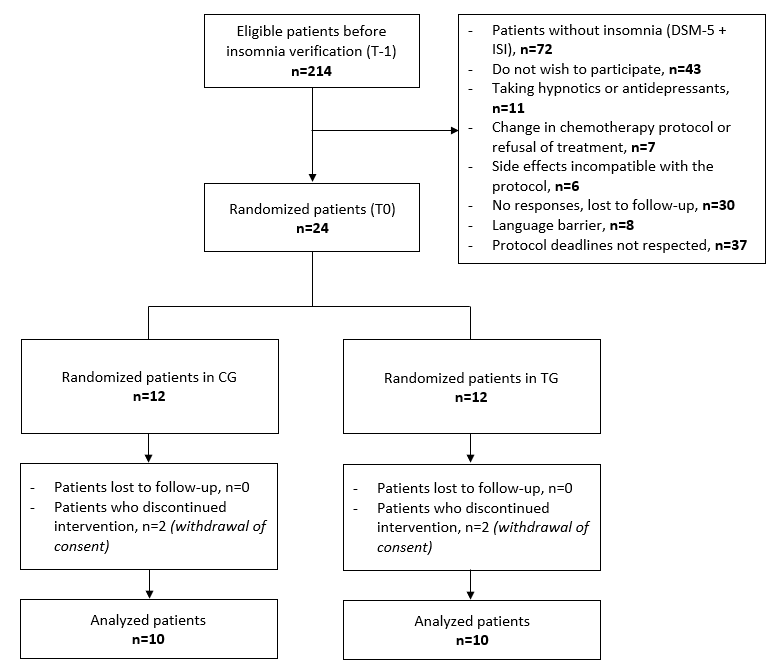


**Figure S1. Flowchart of study protocol**


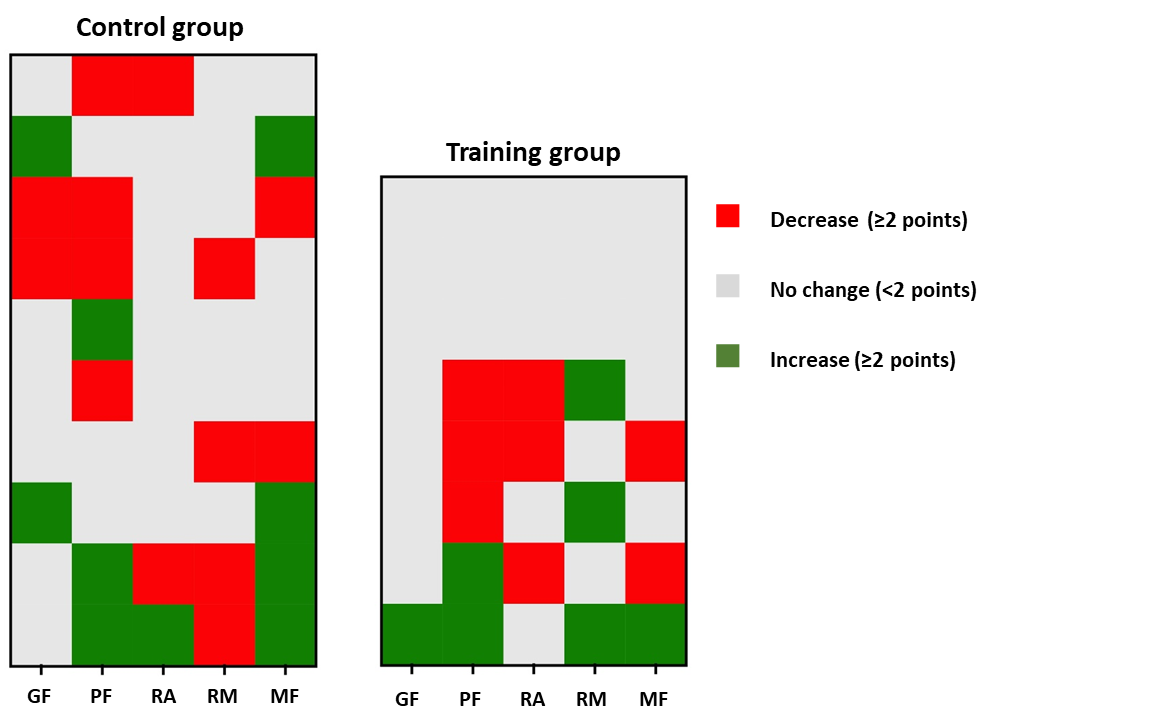


**Figure S2. Individual variations in MFI-20 subscale scores from baseline (T0) to post-intervention (T3) in control and training group**

Colors represent variations exceeding the MCID (≥2 points).

GF: General Fatigue; PF: Physical Fatigue; RA: Reduced Activity; RM: Reduced Motivation; MF: Mental Fatigue.

Each line represents one patient and each column one MFI-20 subscale; red indicates deterioration, grey indicates stability, and green indicates improvement, between T0 and T3.
